# Genomics and proteomics of vertebrate cholesterol ester lipase (*LIPA*) and cholesterol 25-hydroxylase (*CH25H*)

**DOI:** 10.1007/s13205-011-0013-9

**Published:** 2011-08-03

**Authors:** Roger S. Holmes, John L. VandeBerg, Laura A. Cox

**Affiliations:** 1 Department of Genetics, Southwest Foundation for Biomedical Research, San Antonio, TX 78227 USA; 2 Southwest National Primate Research Center, Southwest Foundation for Biomedical Research, San Antonio, TX 78227 USA; 3 School of Biomolecular and Physical Sciences, Griffith University, Nathan, QLD Australia

**Keywords:** Vertebrates, Lipase A, Cholesterol 25-hydroxylase, Cholesterol metabolism

## Abstract

Cholesterol ester lipase (LIPA; EC 3.1.1.13) and cholesterol 25-hydroxylase (CH25H; EC 1.14.99.48) play essential role in cholesterol metabolism in the body by hydrolysing cholesteryl esters and triglycerides within lysosomes (LIPA) and catalysing the formation of 25-hydroxycholesterol from cholesterol (CH25H) which acts to repress cholesterol biosynthesis. Bioinformatic methods were used to predict the amino acid sequences, structures and genomic features of several vertebrate *LIPA* and *CH25H* genes and proteins, and to examine the phylogeny of vertebrate LIPA. Amino acid sequence alignments and predicted subunit structures enabled the identification of key sequences previously reported for human LIPA and CH25H and transmembrane structures for vertebrate CH25H sequences. Vertebrate *LIPA* and *CH25H* genes were located in tandem on all vertebrate genomes examined and showed several predicted transcription factor binding sites and CpG islands located within the 5′ regions of the human genes. Vertebrate *LIPA* genes contained nine coding exons, while all vertebrate *CH25H* genes were without introns. Phylogenetic analysis demonstrated the distinct nature of the vertebrate *LIPA* gene and protein family in comparison with other vertebrate acid lipases and has apparently evolved from an ancestral *LIPA* gene which predated the appearance of vertebrates.

**Electronic supplementary material:**

The online version of this article (doi:10.1007/s13205-011-0013-9) contains supplementary material, which is available to authorized users.

## Introduction

Lysosomal acid lipase or cholesteryl ester hydrolase (also called lipase A or LIPA) (EC 3.1.1.13) catalyses the hydrolysis of cholesterol esters or triglycerides which have been localized within lysosomes following a receptor-mediated endocytosis of low-density lipoprotein (LDL) particles (Goldstein et al. 1975; Anderson et al. 1994; Wang et al. 2008). Inborn errors of metabolism for the human gene encoding this enzyme (*LIPA*) have been described, including Wolman disease (WOD), resulting from a major defect of the gene which leads to a cholesteryl ester storage disease and loss of life, usually within 1 year of age while a second defect of the human *LIPA* gene generates a milder late-onset cholesteryl ester storage disease (CESD) (Beaudet et al. 1977; Burton and Reed 1981; Hoeg et al. 1984).

*LIPA* is localized on chromosome 10 of the human genome and is highly expressed throughout the body, and contains nine coding exons (Koch et al. 1981; Anderson and Sando 1991; Ameis et al. 1994). Several other acid lipase genes, including *LIPF* (encoding gastric triacylglycerol lipase), *LIPJ* (encoding lipase J); and *LIPK*, *LIPM* and *LIPN* (encoding epidermis acid lipases K, M and N), are also located within an acid lipase gene cluster on human chromosome 10 (Bodmer et al. 1987; Deloukas et al. 2004; Toulza et al. 2007). A new acid lipase gene (designated as *Lipo*) has also been recently reported for mouse and rat genomes (Holmes et al. 2010). The human acid lipase gene cluster encodes enzymes with similar sequences which are distinct from the “neutral lipases”, including endothelial lipase (EL), lipoprotein lipase (LPL) and hepatic lipase (HL), which perform specific role in high-density lipoprotein (HDL), LDL and hepatic lipid metabolism, respectively (Wion et al. 1987; Martin et al. 1988; Cai et al. 1989; Ishimura-Oka et al. 1992; Hirata et al. 1999; Jaye et al. 1999).

Cholesterol 25-hydroxylase (CH25H or cholesterol 25-monooxygenase) (EC 1.14.99.38) catalyses the formation of 25-hydroxycholesterol from cholesterol which may serve as a corepressor of cholesterol biosynthetic enymes by blocking sterol regulatory element binding protein processing (Lund et al. 1998). 25-Hydroxysterol is also an activator of gene signalling pathways and an immunoregulatory lipid produced by macrophages to negatively regulate the adaptive immune response in mice (Dwyer et al. 2007; Baumann et al. 2009). CH25H is a member of an enzyme family that utilizes di-iron cofactors to catalyse the hydroxylation of sterol substrates, is encoded by an intronless gene (*CH25H*) located proximally to *LIPA* on human chromosome 10 and is an integral membrane protein located in the endoplasmic reticulum of liver and many other tissues of the body (Lund et al. 1998; Deloukas et al. 2004). Epidemiological studies have suggested that cholesterol metabolism plays a role in Alzheimer’s disease (AD) pathogenesis and several of these genes, including *LIPA* and *CH25H*, have been investigated as possible risk factors for AD (Riemenschneider et al. 2004; Shownkeen et al. 2004; Shibata et al. 2006). Even though a linkage peak was identified within the relevant linkage region on chromosome 10, *LIPA* and *CH25H* gene markers were not significantly associated with susceptibility to AD.

This study describes the predicted sequences, structures and phylogeny of several mammalian and other vertebrate *LIPA* and *CH25H* genes and compares these results for those previously reported for human (*Homo sapiens*) and mouse (*Mus musculus*) *LIPA* and *CH25H* (Koch et al. 1981; Anderson and Sando 1991; Ameis et al. 1994; Lund et al. 1998). Bioinformatic methods were used to predict the sequences and structures for vertebrate LIPA and CH25H and gene locations for these genes, using data from the respective genome sequences. Phylogenetic analyses also describe the relationships and potential origins of vertebrate *LIPA* genes during mammalian and vertebrate evolution in comparison with other acid lipase genes.

## Materials and methods

### Vertebrate lipase and cholesterol 25-hydroxylase gene and protein bioinformatic identification

BLAST (Basic Local Alignment Search Tool) studies were undertaken using web tools from the National Center for Biotechnology Information (NCBI; http://blast.ncbi.nlm.nih.gov/Blast.cgi Altschul et al. 1997). Non-redundant protein sequence databases for several vertebrate genomes were examined using the blastp algorithm, including the chimpanzee (*Pan troglodytes*; The Chimpanzee Sequencing Analysis Consortium 2005), macaque monkey (*Mucaca mulatta*; Rhesus Macaque Genome Sequencing Analysis Consortium 2007) horse (*Equus caballus*; http://www.broadinstitute.org/mammals/horse), cow (*Bos Taurus*; http://www.hgsc.bcm.tmc.edu/projects/bovine/), mouse (*Mus musculus*; Mouse Genome Sequencing Consortium 2002), rat (*Rattus norvegicus*; Rat Genome Sequencing Project Consortium 2004), guinea pig (*Cavia porcellus*http://www.broadinstitute.org/science/projects/mammals-models/guinea-pig/guinea-pig), dog (*Canis familiaris*; http://www.broadinstitute.org/mammals/dog), chicken (*Gallus gallus* International Chicken Genome Sequencing Consortium 2004), and frog (*Xenopus tropicalis*; http://genome.jgi-psf.org/Xentr4/Xentr4.home.html). This procedure produced multiple BLAST “hits” for each of the protein databases which were individually examined and retained in FASTA format, and a record kept was the sequences of predicted mRNAs and encoded LIPA- and CH25H-like proteins. These were derived from annotated genomic sequences using the gene prediction method: GNOMON and predicted sequences with high similarity scores for many of the vertebrate *LIPA* and *CH25H* genes and proteins examined (see Table 1). The orangutan (*Pongo abelii*) and marmoset (*Callithrix jacchus*) genomes were subjected to BLAT (BLAST-Like Alignment Tool) analysis using the human LIPA protein sequence and the UC Santa Cruz genome browser (http://genome.ucsc.edu/cgi-bin/hgBlat) with the default settings to obtain an Ensembl generated protein sequence (Hubbard et al. 2007). A similar BLAT analysis was conducted of the stickleback fish (*Gasterosteus aculeatus*) genome [http://genome.ucsc.edu/cgi-bin/hgBlat] using the frog (*Xenopus tropicalis*) LIPA sequence (see Table 1).

**Table 1:** Vertebrate lipase A (*LIPA*) and cholesterol 25-hydroxylase (*CH24H*) genes and enzymes examined

Lipase gene	Species	RefSeq	GenBank ID	UNIPROT	Amino	Chromosome	Exons	Gene size	p*I*	Subunit	Signal peptide
		Ensembl^a^		ID	acids	location	(strand)	(bps)		(MW)	(cleavage site)
Human *LIPA*	*Homo sapiens*	NM_001127605	BC012287	P38571	399	10:90,964,568-90,997,385	9 (−ve)	32,818	6.42	45,419	1-21 (EG-SG)^c^
Chimp *LIPA*	*Pan troglodytes*	XP_521552^a^	BC012287	P38571	399	10:89,482,403-89,515,834	9 (−ve)	33,432	6.42	45,419	1-21 (EG-SG)^c^
Orangutan *LIPA*	*Pongo abelii*	ENSPPYT00000002953^d^	^b^	^b^	399	10:87,901,737-87,934,555	9 (−ve)	32,819	6.42	45,452	1-21 (EG-SG)^c^
Rhesus *LIPA*	*Macaca mulatta*	XP_001085160^a^	^b^	^b^	399	9:88,805,255-88,839,453	9 (−ve)	34,199	6.39	45,480	1-24 (GG-KL)^c^
Marmoset *LIPA*	*Callithrix jacchus*	4107.004.a^e, f^	^b^	^b^	399	^e^4107:158,060-204,094	9 (+ve)	46,035	6.34	45,424	^b^
Mouse *LIPA*	*Mus musculus*	NM_021460	BC058064	Q9Z0M5	397	19:34,568,473-34,599,332	9 (−ve)	30,860	8.15	45,325	1-25 (VS-AV)^c^
Rat *LIPA*	*Rattus norvegicus*	NM_012732	BC072532	Q64194	397	1:238,468,218-238,497,746	9 (−ve)	29,529	6.30	45,079	1-25 (IS-AV)^c^
Guinea Pig *LIPA*	*Cavia porcellus*	XP_001503012^a^	^b^	^b^	397	1:39,069,159-39,103,381	9 (+ve)	34,223	7.29	46,327	1-22 (RG-KL)^c^
Horse *LIPA*	*Equus caballus*	XP_001503012^a^	^b^	^b^	397	1:39,069,159-39,103,381	9 (+ve)	34,223	7.29	46,327	1-22 (RG-KL)^c^
Cow *LIPA*	*Bos taurus*	NP_001096793^a^	BC146075	^b^	399	26:11,349,737-11,387,245	9 (−ve)	37,509	7.23	45,671	1-23 (SG-WK)^c^
Pig *LIPA*	*Sus scrofa*	NP_001116606^a^	^b^	^b^	399	^b^	^b^	^b^	7.75	45,347	1-19 (HS-EA)^c^
Dog *LIPA*	*Canis familaris*	XP_853280^a^	^b^	^b^	398	26:41,958,963-41,981,595	9 (−ve)	22,633	6.70	45,063	1-19 (RS-EA)^c^
Chicken *LIPA*	*Gallus gallus*	XP_426515^a^	^b^	^b^	402	6:20,252,280-20,262,074	9 (+ve)	9,795	8.44	45,610	1-18 (AG-FT)^c^
Frog *LIPA*	*Xenopus tropicalis*	NM_001015847	BC090136	^b^	404	Sc^e^150:1,826,750-1,838,449	9 (+ve)	11,700	5.81	45,454	1-17 (LT-DD)^c^
Stickleback *LIPA*	*Gasterosteus aculeatus*	ENSGACT00000013219^d^	^b^	^b^	402	V:11,755,530-11,758,732	10 (−ve)	3,203	6.00	45,005	1-17 (LS-GP)^e^
Fruit Fly Lip3	*Drosophila melanogaster*	NM_057983	BT023252	O46108	394	3R:9,195,960-9,197,626	3 (−ve)	1,667	5.40	44,901	1-20 (LA-GS)^c^
*CH25H* Gene											Intragenic (bps)
Human	*Homo sapiens*	NM_003956	BC072430	O095992	272	10:90,956,214-90,957,029	1 (−ve)	816	6.77	31,745	7,539
Rhesus	*Macaca mulatta*	XP_001083208^a^	^b^	^b^	272	9:88,797,797-88,798,612	1 (−ve)	816	6.75	31,850	6,643
Mouse	*Mus musculus*	NP_034020	BC039919	Q9Z0F4	298	19:34,548,723-34,549,616	1 (−ve)	894	7.67	34,672	18,857
Rat	*Rattus norvegicus*	NP_001020586	BC097064	^b^	298	1:238,457,437-238,458,330	1 (−ve)	894	7.07	34,414	9,888
Horse	*Equus caballus*	XP_001503057^a^	^b^	^b^	270	1:39,111,964-39,112,773	1 (+ve)	810	6.73	31,464	8,613
Cow	*Bos taurus*	NP_001068711	BC120312	^b^	270	26:11,336,966-11,337,775	1 (−ve)	810	6.88	31,326	11,972
Dog	*Canis familaris*	XP_543596^a^	^b^	^b^	270	26:41,949,857-41,950,666	1 (−ve)	810	8.88	30,426	8,297
Chicken	*Gallus gallus*	XP_421660^a^	^b^	^b^	274	6:20,415,141-20,415,962	1 (−ve)	822	8.15	32,456	163,682
Frog	*Xenopus tropicalis*	sc.150.119^e, f^	^b^	^b^	272	Sc^e^150:1,998,638-1,999,453	1 (−ve)	816	8.61	31,405	158,189

^a^*RefSeq* The reference mRNA sequence; predicted Ensembl mRNA sequence and GenBank mRNA (or cDNA) IDs are shown (see http://www.ncbi.nlm.nih.gov)

^b^Result not available

^c^Cleavage site predicted for signal peptide at N-termini

^d^Ensembl gene prediction

^e^N-scan gene prediction using the software from the Computational Genomics Lab at Washington University in St. Louis, MO, USA (see http://genome.ucsc.edu)

^f^Contig ID given; UNIPROT refers to UniprotKB/Swiss-Prot IDs for individual LIPA, other acid lipase or CH25H subunits (see http://kr.expasy.org)

*bps* base pairs of nucleotide sequences

*pI* Theoretical isoelectric points the number of coding exons are listed

Sources for LIPA and CH25H sequences were provided by the above sources

BLAT analyses were then undertaken for each of the predicted LIPA and CH25H amino acid sequences using the UC Santa Cruz web browser (http://genome.ucsc.edu/cgi-bin/hgBlat) (Kent et al. 2003) with the default settings to obtain the predicted locations for each of the vertebrate *LIPA* and *CH25H* genes, including predicted exon boundary locations and gene sizes. BLAT analyses were also performed of human *LIPF, LIPJ, LIPK, LIPM* and *LIPN* genes and the mouse *Lipo1*-like gene using previously reported sequences for encoded subunits in each case (see Table 1). Structures for the major human *LIPA* and *CH25H* isoforms (gene splicing variants) were obtained using the AceView website to examine the predicted gene structures using the human *LIPA* and *CH25H* genes to interrogate the database of human mRNA sequences (Thierry-Mieg and Thierry-Mieg 2006) (http://www.ncbi.nlm.nih.gov/IEB/Research/Acembly/index.html?human). Predicted transcription factor binding sites (TFBS) and CpG islands for human *LIPA* and *CH25H* genes were identified using the UC Santa Cruz web browser (http://genome.ucsc.edu/cgi-bin/hgBlat) (Kent et al. 2003).

### Predicted structures and properties for vertebrate LIPA subunits

Predicted structures for vertebrate LIPA subunits were obtained using the SWISS MODEL web tools (http://swissmodel.expasy.org), respectively (Kopp and Schwede 2004). The reported tertiary structure for dog LIPF (Roussel et al. 2002) served as the reference for the predicted vertebrate LIPA tertiary structures, with a modeling range of residues 24–395. Theoretical isoelectric points and molecular weights for vertebrate LIPA and CH25H subunits were obtained using Expasy web tools (http://au.expasy.org/tools/pi_tool.html). SignalP 3.0 web tools were used to predict the presence and location of signal peptide cleavage sites (http://www.cbs.dtu.dk/services/SignalP/) for each of the predicted vertebrate LIPA sequences (Emmanuelsson et al. 2007). The NetNGlyc 1.0 Server was used to predict potential N-glycosylation sites for vertebrate LIPA subunits (http://www.cbs.dtu.dk/services/NetNGlyc/).

### Predicted transmembrane structures for vertebrate CH25H subunits

Predicted transmembrane structures for vertebrate CH25H subunits were obtained using the web server (http://www.cbs.dtu.dk/services/TMHMM-2.0) provided by the Center for Biological Sequence Analysis of the Technical University of Denmark (Krogh and Larsson 2001).

### Phylogenetic studies and sequence divergence

Alignments of protein sequences were assembled using BioEdit v.5.0.1 and the default settings (Hall 1999). Alignment ambiguous regions, including the amino and carboxyl termini, were excluded prior to phylogenetic analysis yielding alignments of 365 residues for comparisons of vertebrate LIPA; human LIPJ; human, mouse and rat LIPF, LIPK, LIPM and LIPN; mouse and rat LIPO;1 and *Drosophila melanogaster* LIP3 sequences (Table 1; Supplementary Table 1). Evolutionary distances were calculated using the Kimura option (Kimura 1983) in TREECON (Van De Peer and de Wachter 1994). Phylogenetic trees were constructed from evolutionary distances using the neighbor-joining method (Saitou and Nei 1987) and were rooted using the *Drosophila melanogaster* LIP3 sequence. Tree topology was reexamined by the boot-strap method (100 bootstraps were applied) of resampling (Felsenstein 1985).

## Results and discussion

### Alignments of vertebrate LIPA amino acid sequences

**Fig. 1:**
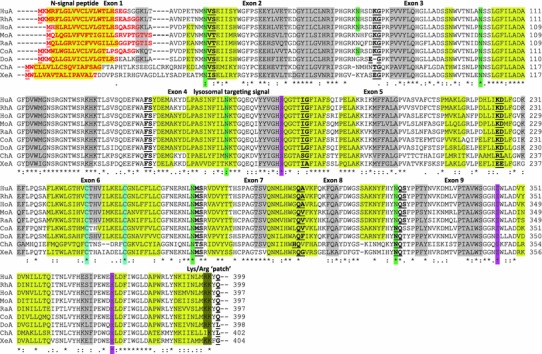
Amino acid sequence alignments for vertebrate LIPA sequences. *HuA* human LIPA, *RhA* rhesus LIPA, *HoA* horse LIPA, *MoA* mouse LIPA, *RaA* rat LIPA, *CoA* cow LIPA, *DoA* dog LIPA, *ChA* chicken LIPA, *XeA* frog LIPA. See Table 1 for sources of LIPA sequences, * identical residues, *colan* 1 or 2 conservative substitutions, *dot* 1 or 2 non-conservative substitutions; residues involved in processing at N-terminus (signal peptide), potential N-glycosylation sites including residues NKT (161–163) which serves as a lysosomal targeting sequence, active site residues Ser174, Asp345, and His374 disulfide bond C residues for human LIPA, helix (human LIPA) or predicted helix; Sheet (human LIPA) or predicted sheet, possible basic amino acid “patch” for lysosomal targeting, *bold underlined* font shows known or predicted exon junctions

The amino acid sequences of derived LIPA subunits are shown in Fig. 1 together with previously reported sequences for human and mouse LIPA (Anderson and Sando 1991; Ameis et al. 1994; Du et al. 1996). Alignments of human LIPA with other predicted vertebrate LIPA sequences showed 64–98% identities, whereas lower levels of identities were observed with human LIPF, LIPJ, LIPK, LIPM and LIPN and with mouse LIPO1 sequences (49–63% identities), and with the *Drosophila melanogaster* LIP3 sequence (38% identity) (alignments of vertebrate LIPA sequences with human and mouse acid lipase gene families are not shown) (Table 2). This comparison suggested that the vertebrate subunits identified were all products of a single gene family (*LIPA*) which is distinct from those previously described for mammalian *LIPF*, *LIPJ*, *LIPK*, *LIPM* and *LIPN* gene families (Bodmer et al. 1987; Toulza et al. 2007; Hirata et al. 1999; Jaye et al. 1999; Wion et al. 1987; Martin et al. 1988) and for a new rodent acid lipase gene family, designated as *Lipo* (Holmes et al. 2010).

**Table 2:** Percentage identities for vertebrate LIPA, human LIPF, LIPJ, LIPK, LIPM and LIPN, mouse LIPO1 and fruit fly (*Drosophila melanogaster*) LIP3 amino acid sequences

Acid lipase gene	Human *LIPA*	Rhesus *LIPA*	Mouse *LIPA*	Chicken *LIPA*	Frog *LIPA*	Human LIPF	Human LIPJ	Human LIPK	Human LIPM	Human LIPN	Mouse LIPO1	Fruit fly LIP3
Human LIPA	100	**98**	**77**	**72**	**69**	61	53	59	63	55	49	38
Rhesus LIPA	**98**	100	**78**	**71**	**69**	60	53	59	63	55	49	37
Mouse LIPA	**77**	**78**	100	**65**	**64**	55	49	55	58	52	47	38
Chicken LIPA	**72**	**71**	**65**	100	**75**	62	54	60	63	55	48	38
Frog LIPA	**69**	**69**	**64**	**75**	100	59	52	58	64	53	50	37
Human LIPF	61	60	55	62	59	100	55	66	55	53	50	37
Human LIPJ	53	53	49	54	52	55	100	57	51	48	46	33
Human LIPK	59	59	55	60	58	66	57	100	57	52	51	32
Human LIPM	63	63	58	63	64	55	51	57	100	54	49	35
Human LIPN	55	55	52	55	53	53	48	52	54	100	44	32
Mouse LIPO1	49	49	47	48	50		46	51	49	44	100	35
Fruit Fly LIP3	38	37	38	38	37	37	33	32	35	32	35	100

Numbers show the percentage of amino acid sequence identities. Numbers in *bold* show higher sequence identities for vertebrate LIPA sequences

The predicted amino acid sequences for these vertebrate LIPA subunits were all of similar length (397–404 residues) and shared many (~34%) of identically aligned residues (Fig. 1; Table 1). In addition, key residues previously described for human gastric acid lipase (LIPF) (Roussel et al. 1999) and for human LIPA (Zschenker et al. 2004) involved in catalysis and maintaining enzyme structure were conserved. Those retained for catalytic function included the active site residues involved with the charge relay system (human LIPA residue numbers used) (Ser174; Asp345; His374); the active site motif (Gly-Xaa-Ser-Yaa-Gly) (residues 172–176); and cysteine residues forming a disulfide bond (Cys248/Cys257) to support the enzyme’s structure.

The hydrophobic N-terminus signal peptide function (residues 1–18 for human LIPA), the mannose-6-phosphate containing N-glycosylation site (residues 161–163: Asn-Lys-Thr) and the C-terminal sequence (residues 396–397 Arg-Lys for human LIPA), which may contribute to the lysosomal targeting of LIPA (Sleat et al. 2006), have been retained or underwent conservative substitution(s) for all vertebrate LIPA sequences examined (with the exception of the chicken LIPA C-terminal sequence) (residues 399–400 Ile-Lys) (Fig. 1). Two of the other high probability N-glycosylation sites for human LIPA (Asn36-Val37-Ser38 and Asn273-274Met-275Ser) were retained for all of the vertebrate LIPA sequences examined, while another was conserved for some vertebrate LIPA sequences (Asn72-His73-Ser74) (Fig. 1; Table 3). There were species differences observed for the theoretical isoelectric points (*pI*) of the vertebrate LIPA subunits, with predicted higher values (*pI* values >8) for mouse and chicken LIPA (Table 1).

**Table 3:** Predicted N-glycosylation sites for vertebrate LIPA subunits

Vertebrate LIPA protein	Species	Site 1	Site 2	Site 3	Site 4	Site 5	Site 6	Potential N-glycosylation sites	High probability sites (>0.75)	Lower probability sites (0.5–0.74)
Human	*Homo sapiens*	**36NVS**	**72NHS**	101NSS	**161NKT**	**273NMS**	321NQS	6	**4**	2
Rhesus	*Macaca mulatta*	**36NVS**	**72NHS**	101NSS	**161NKT**	**273NMS**	321NQS	6	**4**	2
Mouse	*Mus musculus*	**34NVT**		99NSS	**159NKT**	**271NMS**	319NQS	5	**3**	2
Rat	*Rattus norvegicus*	**34NVT**		99NSS	**159NKT**	**271NMS**	319NQS	5	**3**	2
Horse	*Equus caballus*	**34NVS**		99NSS	**159NKT**	**271NMS**	319NQS	5	**3**	2
Cow	*Bos taurus*	**36NVS**	**72NRS**	101NSS	**161NKT**	**273NMS**	321NQS	6	**4**	2
Dog	*Canis familiaris*	**36NVS**		100NSS	160NKT	**272NMS**	320NQT	5	**2**	3
Chicken	*Gallus gallus*	43NVS		107NNS	167NKT	**277NTS**	324NQT	5	**1**	4
Frog	*Xenopus tropicalis*	43NIS		107NNS		**279NMS**	326NQT	4	**1**	3
Fish	*Gasterosteus aculeatus*	**39NIS**				**277NMT**	324NQS	3	**2**	0

Numbers refer to amino acids in the LIPA sequences, including *N* asparagine, *K* lysine, *I* isoleucine, *M* methionine, *H* histidine; *S* serine, *R* arginine, *T* threonine, *Q* glutamine, and *V* valine. Note that there are six potential sites identified. High (*in bold*) and lower probability N-glycosylation sites were identified using the NetNGlyc 1.0 web server (http://www.cbs.dtu.dk/services/NetNGlyc/)

### Alignments of vertebrate CH25H amino acid sequences

**Fig. 2:**
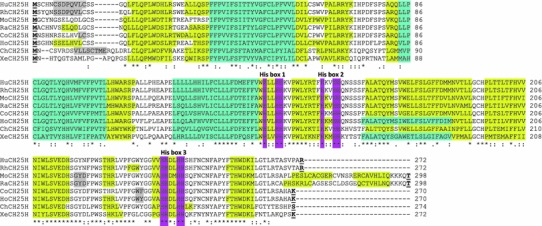
Amino acid sequence alignments for vertebrate CH25H sequences. *HuCH25H* Human CH25H, *RhCH25H* rhesus CH25H, *MoCH25H* mouse CH25H, *RaCH25H* rat CH25H, *CoCH25H* cow CH25H, *HoCH25H* horse CH25H, *ChCH25H* chicken CH25H, *XeCH25H* frog CH25H. See Table 1 for sources of CH25H sequences. * identical residues; *colon* 1 or 2 conservative substitutions, *dot* 1 or 2 non-conservative substitutions, histidine residues active site boxes 1, 2 and 3, predicted helix, predicted sheet, predicted transmembrane regions, *bold underlined* font shows known or predicted exon junctions (single exon *CH25H* genes observed in each case)

**Fig. 3:**
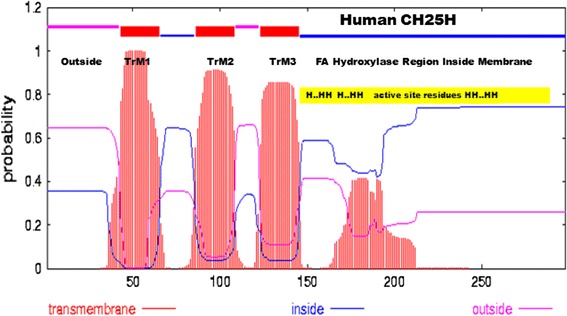
Predicted locations for transmembrane regions for human CH25H. The graph shows probability (0–1 on *y* axis) of transmembrane regions (TrM1, TrM2 and TrM3 shown in *red*) for the human CH25H amino acid sequence (on *x* axis). Predicted outside membrane CH25H residues are shown in *red*; predicted inside membrane CH25H residues are shown in *blue*, predicted positioning of the three histidine active site boxes are shown as *H..HH* or *HH..HH* and are localized inside the membrane

Amino acid sequence alignments of derived CH25H subunits are shown in Fig. 2 together with previously reported sequences for human and mouse CH25H (Lund et al. 1998; Zhao et al. 2005). Most of the vertebrate CH25H sequences were 270–274 amino acid residues in length, with the exception of mouse and rat CH25H which exhibited extended C-termini, and contained 298 residues. Three histidine boxes reported for human CH25H (Lund et al. 1998) have been conserved for all vertebrate CH25H sequences examined, including box 1 (Trp-His-Leu/Val-Leu-Val-His-His) for residues 142–148; box 2 (Phe/Ile-His-Lys-Val/Met/Leu-His-His) for residues 157–162; and box 3 (His–His-Asp-Leu/Met-His-His) for residues 238–244 (Fig. 2). These have been previously shown to be essential for CH25H catalytic activity and bind the iron atoms which assist in the hydroxylation reaction (Fox et al. 1994). Predicted transmembrane structures for vertebrate CH25H are also shown (Fig. 2), for which three such regions were predominantly retained for the sequences examined. Figure 3 examines in more detail the predicted positioning of the three transmembrane domains within the human CH25H sequence which suggest that the N-terminus commences outside the endoplasmic reticulum, and that the three active site histidine boxes are localized inside the membrane of the endoplasmic reticulum, where CH25H catalysis is likely to take place.

### Comparative vertebrate *LIPA* and *CH25H* genomics

**Fig. 4:**
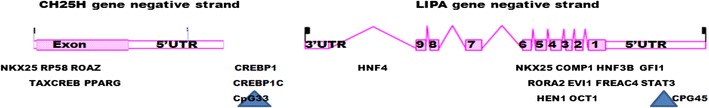
Gene structures and tandem locations for the human *CH25H* and LIPA genes on chromosome 10 derived from the AceView website http://www.ncbi.nlm.nih.gov/IEB/Research/Acembly/, (Reimenschneider et al. 2004); isoform variant LIPAb and CH25H mRNAs are shown with capped 5′- and validated 3′-ends for the predicted sequences, predicted exon regions are shaded, note that CH25H is predicted as a single exon gene, 5′UTR and 3′UTR refer to untranslated 5′ and 3′ regions, respectively, predicted transcription factor binding sites are shown. *NKX25* homeobox protein 2.5, *RP58* transcriptional repressor RP58, *ROAZ* zinc finger protein 423, *TAXCREB*, *CREBP1* and *CREBP1C* cyclic-AMP responsive element-binding proteins, *PPARG* peroxisome proliferator-activated receptor gamma, *HNF4* hepatocyte nuclear factor 4-alpha, *COMP1* muscle specific transcription enhancer, *HNF3B* hepatocyte nuclear factor 3-beta, *GFI1* zinc finger protein GFI1, *RORA2* alpha orphan nuclear receptor, *EVI1* zinc finger protein EVI1, *FREAC4* forkhead box protein, *STAT3* identified in the promoters of acute-phase genes, *HEN1* helix-loop-helix protein 1, and *OCT1* transcription factor that binds to the octomer motif, predicted locations for CpG islands (CPG45; CPG33) are shown by *shaded triangles*

The AceView web browser defines the human *LIPA* gene by 1443 GenBank accessions from cDNA clones derived from spleen, brain, liver and many other tissues and reports a high expression level (~4.9 times the average human gene) (http://www.ncbi.nlm.nih.gov/IEB/Research/Acembly/) (Thierry-Mieg and Thierry-Mieg 2006). Human *LIPA* transcripts included 22 alternatively spliced variants, which differed by truncations of the 5′ or 3′ ends, the presence or absence of 10 cassette exons, or had overlapping exons with different boundaries. Of these, five encoded complete proteins, including isoform *LIPAb* (RefSeq NM_00235) shown in Fig. 4. The predicted 38.47 kb sequence contained ten premessenger exons and nine coding exons as well as several transcription factor binding sites (TFBS) and a CpG island (designated as CpG45) within the 5′-untranslated region for the human *LIPA* gene (Fig. 4). Figure 1 compares the locations of the intron–exon boundaries for the vertebrate *LIPA* gene products examined. Exon 1 corresponded to the encoded signal peptide in each case, and exon 4 encoded the lysosomal targeting sequence (for human *LIPA* residues 161–163 Asn-Lys-Thr) (Sleat et al. 2006). There is identity or near identity for the intron–exon boundaries for each of the vertebrate *LIPA* genes suggesting conservation of these exons during vertebrate evolution.

In contrast to human *LIPA*, the human *CH25H* gene is defined by only 29 GenBank accessions for the AceView web browser from cDNA clones derived from 14 tissues including pancreas, brain and lung and showed a reduced expression level (~25% of the average human gene) (http://www.ncbi.nlm.nih.gov/IEB/Research/Acembly/) (Thierry-Mieg and Thierry-Mieg 2006). Moreover, a single human *CH25H* transcript was recorded covering 1.7 kb of sequence which was intronless and contained a large 5′ untranslated sequence proximally located near the 3′ region of the *LIPA* gene (Fig. 4), which is consistent with a previous report (Lund et al. 1998). The human CH25H genome sequence contained several predicted TFBS sites and a CpG island (CpG33) located in the intragenic region (~7.5 kb) separating the human *CH25H* and *LIPA* genes on chromosome 10. Of particular significance were the CREB (cyclic-AMP response element-binding) binding sites, which may play a role in driving expression from the *CH25H* promoter (Watters and Nourse 2009). The close proximal location of these genes was also observed for all other mammalian genomes examined (<20 kb) (Table 1), while chicken (*Gallus gallus*) and frog (*Xenopus tropicalis*) *LIPA* and *CH25H* genes were more distantly located (~160 kb). CpG islands were observed in the human *LIPA*-*CH25H* intragenic region and in the 5′-untranslated *LIPA* region which may reflect roles for these CpG islands in up-regulating gene expression (Saxonov et al. 2006), given their colocation with the *LIPA* and *CH25H* promoters.

### Secondary and tertiary structures for vertebrate *LIPA* sequences

**Fig. 5:**
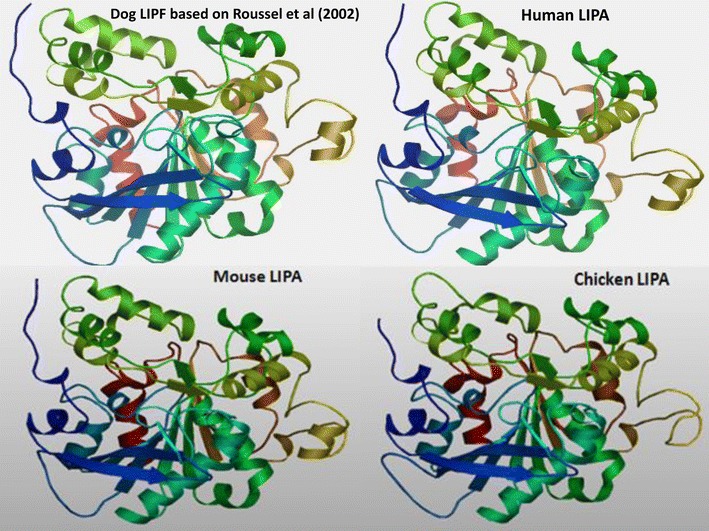
Comparison of predicted three-dimensional structures for human, mouse and chicken LIPA subunits with the known structure for dog LIPF (from Roussel et al. 2002). Predicted 3D structures were obtained using the SWISS MODEL (http://swissmodel.expasy.org/workspace/index.php) web site and the predicted amino acid sequences for vertebrate LIPA subunits (see Table 1). The *rainbow color code* describes the 3D structures from the N- (*blue*) to C-termini (*red color*). The structures are based on the known 3D structures for dog LIPF (from Roussel et al. 2002) (with a modeling range of residues 24–395 for human, mouse and chicken LIPA)

Figure 1 shows the secondary structures predicted for vertebrate LIPA sequences. Similar α-helix β-sheet structures were observed for all of the vertebrate LIPA subunits examined, particularly near key residues or functional domains, including the α-helix within the N-terminal signal peptide, the β-sheet and α-helix structures surrounding the active site Ser174 (for human LIPA), the α-helix enclosing the lysosomal targeting signal residues (Asn-Lys-Thr residues 161–163 for human LIPA) and the C-terminal α-helix containing the basic amino acid residue ‘patch’ (residues 396–397 Arg-Lys), which may contribute to LIPA lysosomal microlocalization (Sleat et al. 2006). Predicted LIPA secondary structures, however, may not fully reflect structures in vivo and serve only as a guide to the comparative structures for vertebrate LIPA subunits. The predicted tertiary structures for human, mouse, cow and chicken LIPA were sufficiently similar to the previously reported dog LIPF (gastric acid lipase) structure (Roussel et al. 2002) (Fig. 5) but were based on incomplete sequences for human, mouse and cow LIPA (residues 24–395 for human LIPA). These results suggested that the major structural features for human LIPA recently reported (Roussel et al. 1999) resemble those for other vertebrate LIPA proteins, as well as for the dog gastric LIPF structure.

### Phylogeny of vertebrate *LIPA* and other human acid lipase genes and proteins

**Fig. 6:**
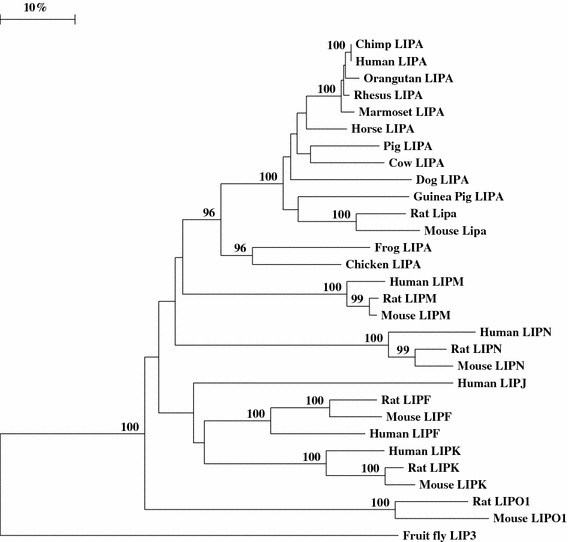
Phylogenetic tree of vertebrate LIPA, other human, mouse and rat acid lipases and *Drosophila melanogaster* LIP3 sequences. The tree is labeled with the lipase gene family number and the species name. Note the separation of the mammalian LIPF, LIPJ, LIPK, LIPM, LIPN and LIPO family sequences from the vertebrate LIPA family cluster. The *Drosophila melanogaster* LIP3 sequence was used to root the tree. A genetic distance scale is shown. The number of times a clade (sequences common to a node or branch) occurred in the bootstrap replicates are shown. Replicate values of 90 or more are highly significant (shown in *bold*). 100 bootstrap replicates were performed in each case

Phylogenetic trees (Fig. 6) were constructed from alignments of vertebrate LIPA-like amino acid sequences with human LIPJ, human; mouse and rat LIPF, LIPJ, LIPK, LIPM and LIPN; and mouse and rat LIPO1 sequences (for further details see Supplementary Table 1; and Holmes et al. 2010). The dendrogram was rooted using a *Drosophila melanogaster* LIP3 sequence (Pistillo et al. 1998) and showed clustering of all of the LIPA-like sequences which were distinct from the other human and mouse acid lipase gene families. The results were consistent with these acid lipase genes being products of gene duplication events prior to vertebrate evolution, particularly for the *LIPA* gene family, which is of apparent ancient origin of more than 500 million years ago (Donoghue and Benton 2007). Table 2 summarizes the percentages of identity for these enzymes and shows that vertebrate LIPA sequences are ≥64% identical which is in comparison with the 44–63% identities observed comparing sequence identities between acid lipase families. In addition, more closely related species showed higher levels of sequence identity for LIPA, such as the primate species (human and rhesus monkey) which were 98% identical, as compared with the bird (chicken) and human LIPA sequences, with 72% identical sequences.

## Conclusions

Based on this report, we propose that an acid lipase primordial gene predated the appearance of vertebrates and underwent successive gene duplication events generating at least seven acid lipase gene families, namely *LIPA* (encoding lysosomal lipase), *LIPF* (encoding gastric lipase) and five other gene families (*LIPJ*, *LIPK*, *LIPM*, *LIPN* and *LIPO*), which have been retained as separate vertebrate gene families for more than 500 million years. In addition, it is likely that the *LIPA* gene family has been conserved throughout vertebrate evolution to serve a major role as an acid lysosomal lipase, given the conservation of key residues and lysosomal targeting sequences for vertebrate *LIPA* proteins.

## Electronic supplementary material

Below is the link to the electronic supplementary material.

Supplementary Table 1 Human, Mouse and Rat Acid Lipases Genes and Enzymes Examined RefSeq: the reference mRNA sequence; ¹predicted Ensembl mRNA sequence; and GenBank mRNA (or cDNA) IDs are shown (see http://www.ncbi.nlm.nih.gov); ²result not available; UNIPROT refers to UniprotKB/Swiss-Prot IDs for individual acid lipase subunits (see http://kr.expasy.org); bps refers to base pairs of nucleotide sequences; the number of coding exons are listed.(XLS 36 kb)
